# Creative therapy in health and disease: Inner vision

**DOI:** 10.1111/cns.14266

**Published:** 2023-06-12

**Authors:** Radwa Khalil, Vida Demarin

**Affiliations:** ^1^ School of Business, Social and Decision Sciences Constructor University Bremen Germany; ^2^ International Institute for Brain Health Zagreb Croatia

**Keywords:** brain plasticity, creative therapy, de novo abilities, dementia, experience flow, kinesthetic creativity, musical creativity, visuospatial creativity

## Abstract

Can we better understand the unique mechanisms of de novo abilities in light of our current knowledge of the psychological and neuroscientific literature on creativity? This review outlines the state‐of‐the‐art in the neuroscience of creativity and points out crucial aspects that still demand further exploration, such as brain plasticity. The progressive development of current neuroscience research on creativity presents a multitude of prospects and potentials for furnishing efficacious therapy in the context of health and illness. Therefore, we discuss directions for future studies, identifying a focus on pinpointing the neglected beneficial practices for creative therapy. We emphasize the neglected neuroscience perspective of creativity on health and disease and how creative therapy could offer limitless possibilities to improve our well‐being and give hope to patients with neurodegenerative diseases to compensate for their brain injuries and cognitive impairments by expressing their hidden creativity.

## Graphical abstract

Implementing an effective creative therapy in health and disease requires an investigation of brain plasticity in cognitive control, which may have varying effects on creativity domains such as musical, visuospatial, and kinesthetic creativity, based on the associated mental operations involved.

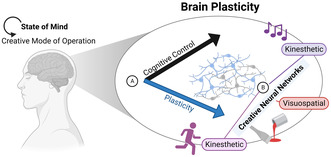

## 1 INTRODUCTION

Creativity makes invisible (novel and imaginative ideas) visible, and every individual possesses a level of creativity that varies in quality and degree. This creative act allows us to perceive the world differently, find hidden patterns, and construct connections between seemingly unrelated events to generate extraordinary output.^1^, ^2^, ^3^, ^4^ Following a summary of the fundamental theories on creativity conceptualization, we will discuss the various hypotheses that attempt to explain the mental processes involved in creativity. We shed light on the significance of skill development in enhancing creativity in health and disease. Therefore, we contend that brain plasticity associated with mental operations of creativity in various domains symbolizes the latent power of creative therapy in art, writing, music, and kinesthetic creativity (i.e., dance and sports). Through case studies, we demonstrate the ignored importance of creative therapy.

As discussed by **Rollo May** in his book, *The Courage to Create* ^5^, creativity brings to our awareness what has previously been hidden, opening new perspectives and visualizing what is invisible. Thus, the experience of enhanced consciousness is joyful. Fifty‐eight years ago, **Ellis Paul Torrance** began investigating creativity, especially in children.^6^, ^7^, ^8^, ^9^ He emphasized the role of the teacher and the significance of developing children's creative potential in the classroom. **Robert Sternberg** and his colleagues have also initiated considerable research on creativity assessment, who in 1991 established the *Investment Theory of Creativity and Development.* ^10^, ^11^ The innate drivers for realizing one's unique potential explain the demand to be creatively identified as part of human self‐actualization within ***Maslow**'s hierarchy* of needs.^12^

**FIGURE 1:**
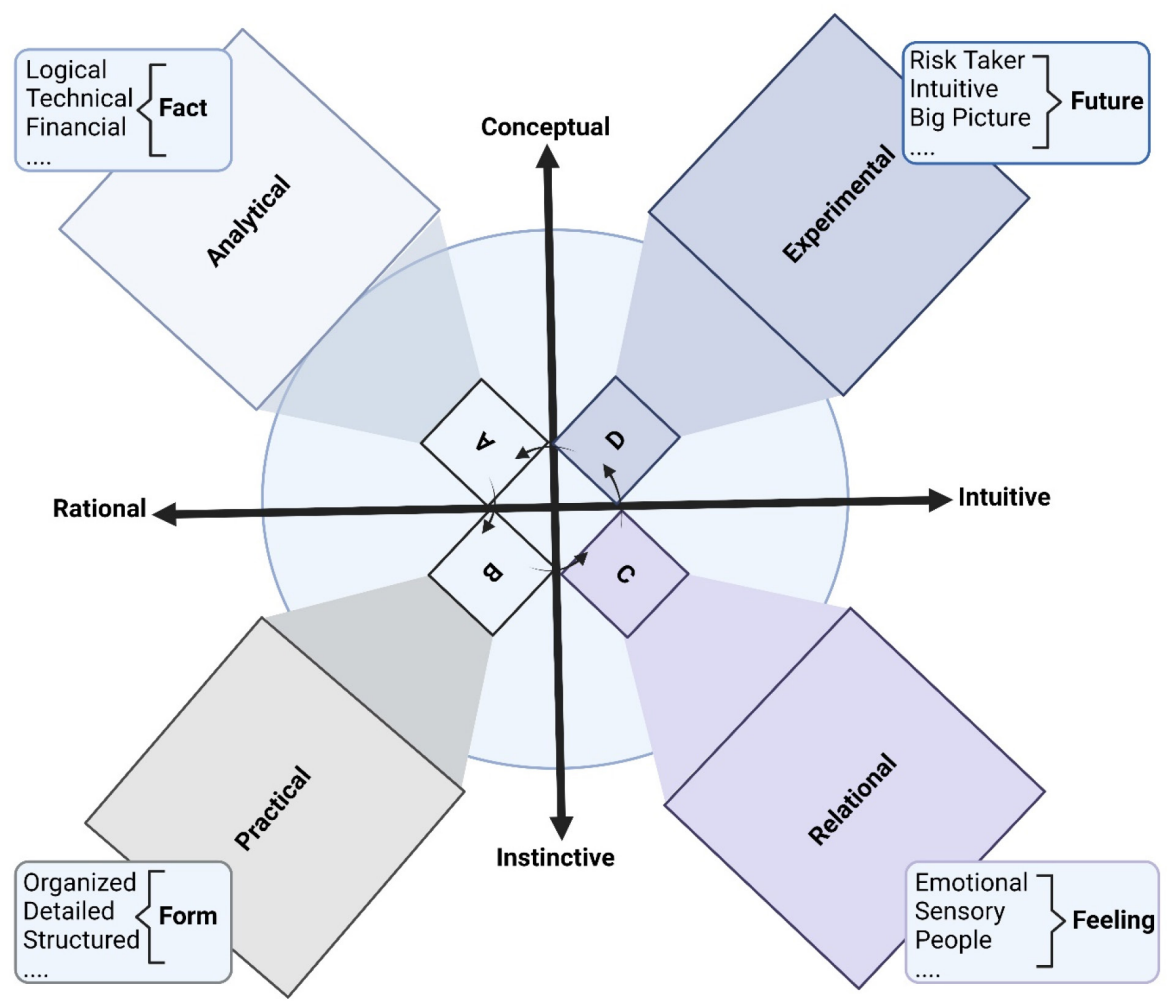
Herrmann brain dominance instrument. This graph illustrates four specialized clusters of mental activity. The four quadrants are divided based on conceptual, instinctive, initiative, and relational axes. A/Facts, Analytical: logical, technical, financial, fact‐based, etc.; B/Form, Practical: organized, detailed, structured, etc.; C/Feelings, Relational: emotional, sensory, etc.; and D/Future, Experimental: risk taker, intuitive, etc.

After more than 30 years of experimenting with creativity and individual differences, **Csikszentmihalyi** ^13^, ^14^ stated that it is the notion of complexity that makes creative individuals' personalities distinct from others. According to **Csikszentmihalyi**, creative individuals show tendencies of thought and action that, in most individuals, are segregated, and these thoughts and actions feature contradictory extremes; instead of being “single,” they are “multitudinous.” One possibility to interpret this complex phenomenon of creativity and how it originated and expressed is to investigate the brain using the brain‐process and process‐brain approaches. **Ned Herrmann** developed his concept of the *Whole‐Brain Thinking System* (WBTS),^15^ which includes four types of thinking, each roughly corresponding to one brain structure. This concept merges *the left and right brain hemisphere theories* with ***Paul MacLean**'s triune theory* ^16^ into a novel model of thinking styles. According to the WBTS concept, each person has a preferred thinking style, and others also have their preferred thinking styles; every person has a signature of his/her preferred thinking style(s). Four patterns in how the brain perceives and processes information led **Herrmann** to develop his concept. He constructed a measuring tool for observing these differences called the *Herrmann Brain Dominance Instrument* (Figure 1).

**Herrmann** ^15^ explained his four specialized clusters of mental activity: *A/Facts—Goals‐driven*: logical, analytical, fact‐based, quantitative intelligence; *B/Form—Results‐driven*: structural–operational, sequential, organized, detailed planned intelligence; *C/Feelings—People‐driven*: social‐relational intelligence, interpersonal, feeling‐based, emotional intelligence; and *D/Future—Vision‐driven*: conceptual‐creative, intuitive, integrating, synthesizing intelligence. Therefore, creativity is personal; we should maintain our creative space at work and within the family and universally unleash our enormous creative capability.

Another reason for the complexity of pinning down creativity stems from the fact that there are various creativity domains, including musical, literary, visual, kinesthetic, and scientific creativity. These domains are strongly interacting and intertwined, reflecting diverse views and opinions; for instance, creative architecture and designs represent a combination of both artistic and scientific creativity.^2^ Also, there is a distinctive difference between art, science, and humor,^17^ spontaneous and deliberate modes of creativity,^18^, ^19^ and problem‐solving and expression.^20^ From the neuroscientific perspective, theoretical frameworks that posit brain‐based differences in artistic versus scientific creativity are scarce.^21^ There is still considerable debate about these types of creativity and how best to conceptualize their commonalities and distinctions within a single viable framework.^2^ Nonetheless, advancements in cognitive neuroscience concerning creativity offer additional promises to unravel this debate.

The recapitulated purpose of cognitive neuroscience of creativity is to clarify how such a multitude of creative cognition could be mapped into specific features of brain regions (structural mapping) or specific activities within brain regions (functional mapping).^2^, ^20^ Though earlier times witnessed the mapping of functions to “locally separated brain regions,” the trajectory has today shifted toward “integrated activity of large‐scale, distributed networks of brain regions”.^22^, ^23^, ^24^, ^25^ The physiological approach to understanding creativity has a very long tradition, beginning in the 1940s when researchers examined the consequences—positive, negative, or unchanged—of prefrontal leucotomies,^26^, ^27^ a controversial procedure that involved severing the connections to and from the prefrontal cortex (PFC). The 1970s and 1980s witnessed the use of electroencephalography (EEG) to measure the type and pattern of brain activity exhibited during creative thinking.^28^, ^29^, ^30^

Fast forward to the present day, and the latest trend in the physiological approach to understanding creativity is to characterize the mechanisms underlying creativity in the context of brain networks.^2^, ^25^, ^31^, ^32^, ^33^, ^34^, ^35^, ^36^, ^37^ The range of paradigms, tasks, and techniques that have been used in the physiological approach has led to a diverse constellation of findings, and these have been used in different ways to explain the basis of creativity from the perspective of the nervous system.^2^, ^20^, ^37^, ^38^, ^39^ The physiological approach to creativity has explored the relationship of creative engagement to the activity of the peripheral nervous system, such as enhanced sympathetic cardiac activity during divergent thinking (DT)^40^, ^41^ and increased pupil dilation during music‐induced aesthetic “chills”.^42^ As most empirical investigations and theories concerning such physiological correlates of creative thinking are focused on the central nervous system and the brain in particular, these can fall into one of two categories: local and global.^2^

Local explanations derive from specific physiological markers or indices to characterize how the brain facilitates particular mental operations relevant to creative thinking (e.g., analogical reasoning, conceptual expansion). Specificity is, therefore, the doctrine of local explanations regarding zoning in on the particular creativity‐relevant cognitive processes under study and the engagement of select brain regions or activity patterns.^31^, ^43^ However, the correlations between the mind's and nervous system's operations are far from linear.

**FIGURE 2:**
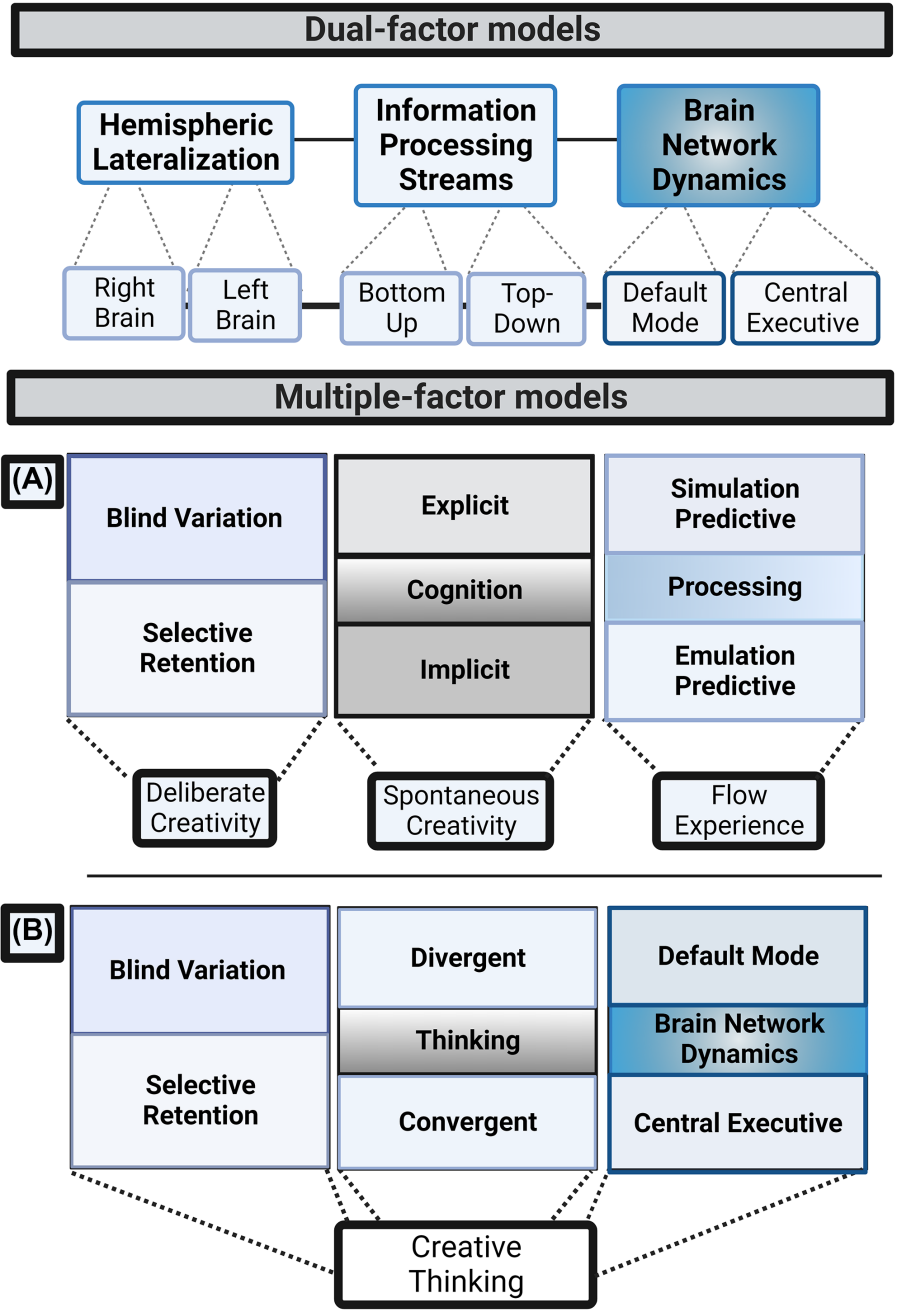
Dual and multiple factor models. The dual models of creativity consider hemispheric lateralization (right vs. left brain), information processing streams (bottom‐up vs. top‐down approaches), and brain network dynamics (default vs. central executive modes). Multiple‐factor models reflect the parallel machinery of three (or more) systems that operate in a union. The two multiple‐factor models that have been proposed hypothesize the information processing mechanisms underlying (A) the many operations underlying various types of creative insight (*Dietrich's evolutionary predictive perspective*) or (B) different aspects of the creative‐thinking process in general (*Jung's evolutionary brain networks perspective*).

Global explanations regarding the physiological basis of creativity that center on the mechanisms of large and widely dispersed systems in the brain are referred to as “global”. This global explanation has two key common features: it typically draws on dualistic or triadic models of brain functions, and these functions map into large brain networks (e.g., the default mode network (DMN) and the central executive network (CEN)) or minimally differentiated large brain structures (e.g., the right versus left hemisphere). More recent models that propose global explanations of creative thinking have recognized the necessity of considering multiple factors when attempting to comprehend the neural basis of creativity. As a result, the concept of multiple‐factor models emerged to go beyond the dual models of creativity by considering the simultaneous operations of three (or more) systems that function in conjunction. The two multiple‐factor models that have been proposed to postulate the information processing mechanisms underlying (a) different aspects of the creative thinking process in general (*Jung's evolutionary brain networks perspective*) or (b) the many operations underlying different types of creative insight (*Dietrich's evolutionary predictive perspective*) are shown in Figure 2.

## 2 CREATIVE MENTAL OPERATIONS

The mental operations provide cognitive explanations for developing creative ideas and applying them in the frameworks of motivation, perception, learning, thinking, and communication.^44^ The correlation between the brain process approach and the neuroscientific perspective is readily apparent, given that neuroscience is concerned with investigating the structure and function of the human nervous system. Despite the singular nature of the term “process,” it would be inaccurate to view the creative process as a singular or unified entity in any manner. The term in question serves as a concise descriptor for multifaceted arrays of cognitive, motivational, and emotional processes implicated in various mental activities such as perception, memory, imagination, appreciation, reasoning, strategizing, etc.^45^ Several mental processes related to creativity have been studied psychologically, such as conceptual expansion, creative imagery, overcoming knowledge constraints, analogical reasoning, and metaphor processing.^46^, ^47^, ^48^, ^49^ Conquering the hindering influence of prominent information (overcoming knowledge constraints) for creating novel and useful ideas requires integrating basic abstract elements (creative imagery). This novel idea could be utilized in one domain to tackle problems in another (analogical reasoning) or gain a more profound conceptual comprehension of a specific phenomenon in diverse contexts (metaphor processing)^2^, ^43^ .

Conceptual expansion is a major part of creative thinking; it means broadening our ideas or going beyond the limits of our existing conceptual frameworks of semantic information. The “aha” experience that accompanies solving analytical problems is also a part of creative mental operation (i.e., insight; for a review, see^50^). All of us have encountered situations where we have reached an impasse and can not find a solution to a problem. At this moment, frustration arises, and a resolution suddenly emerges into an individual's awareness, apparently from an unknown source. A sudden realization or epiphany, colloquially known as an “eureka” moment or “aha” experience, is what marks the insight phenomenon.^50^ This experience facilitates the expansion of pre‐existing conceptual knowledge structures to incorporate novel elements (i.e., conceptual expansion). The relevance of insight lies in its ability to emerge involuntarily upon reaching a solution, which is achieved by overcoming functional fixedness and engaging in a perspective shift.

Therefore, these mental operations are cooperative and cannot be operated on or processed separately. Here, it is possible that the phenomenology of “surprise” is a factor to be considered among the previously mentioned elements of creative mental operations. In emphasizing that several mental (cognitive) operations are involved in creative thinking and not only one, which can be assessed by examining normative cognitive processes under explicitly generative conditions, this approach duly acknowledges the multifaceted nature of creativity. Therefore, the nature of the context in which creative idea generation is called for (the creativity task being employed and the mental operations involved during task engagement) cannot be disregarded. ^51^ For instance, the creative process of “conceptual integration” or “conceptual blending” is central to the poetics of literature, as this mental operation allows for the generation of novel meaning out of existing or old concepts and, as such, has a role to play in semantics, grammar, meaning, discourse, humor, poetry, and so on.^52^

Clustering the mental operations of creative cognition often examined in isolation to reveal the information processes and the neural mechanisms underlying creativity, is one of the major challenges in creativity research.^2^, ^18^, ^19^, ^20^ Most conclusions are built on the basis that these diverse operations are mutually exclusive and do not interact; in fact, the opposite is the case. While engaging in creative pursuits in any domain of human endeavor, these diverse operations of creative cognition are orchestrated together and reflect the underlying integrated dynamic.^2^, ^31^, ^32^, ^36^, ^43^, ^53^, ^54^ This dynamic interplay between both brain hemispheres through specific nodes (network hubs)^33^, ^35^, ^37^, ^38^, ^55^ remodels the mental workspace, the deliberate and spontaneous modes, and the flow of experience.^18^, ^56^, ^57^, ^58^ In the subsequent sections, we will emphasize these underpinning dynamics.

### 2.1 Cross‐hemispheric communication

The persistence of the creative right brain's concept has a strong resonance, especially considering that even the earliest advocates of lateralization research highlighted the significant role of both brain hemispheres in creativity.^59^, ^60^, ^61^ The right hemisphere is specialized for metaphoric thinking, playfulness, solution‐finding, and synthesizing, and it is the center of visualization, imagination, and conceptualization; however, the left hemisphere is still required for artistic work to achieve balance, which partly suppresses the creative states of the right hemisphere.^4^ Thus, drawing a simple inference about the nature of hemispheric differences (i.e., right vs. left) as a function of creativity is unfeasible. Instead, regions in both hemispheres are involved in creativity, even if the information processing about certain aspects of creative cognition may be biased regarding hemispheric lateralization.^62^, ^63^, ^64^

This emphasis is evident among current opinions,^65^, ^66^, ^67^, ^68^ with many studies pointing to the necessity of considering individual differences in creativity further.^35^, ^69^, ^70^ As an example, more creative individuals exhibit bilateral hemispheric activity; in contrast, less creative individuals show more right‐lateralized activity.^71^, ^72^

Brain imaging studies revealed that engaging in creative activities enhances hemispheric connectivity, and individuals with a larger corpus callosum tend to score higher on measures of creativity, which suggests that effective communication and integration of information between and within both brain hemispheres maybe crucial for creative thinking.^59^, ^73^ Indeed, when estimating differences in the structure of brain networks using Bayesian procedures, it has been shown that highly creative individuals demonstrate a higher tendency to form interhemispheric connections.^74^ Recent evidence suggests that both hemispheres are essential in integrating information and thinking creatively, mainly when communicating, and that static and dynamic functional connectivity support the configuration of brain networks for creative cognition.^75^

### 2.2 The dynamic interplay between the three distinctive creative network models

Network theories of psychological functions are increasingly more dominant and influential than modular theories, which also apply to the neuroscience of creativity.^2^, ^20^, ^76^ Unsurprisingly, during the creative process, there is a dynamic interplay of our unconscious and conscious processing that engages emotional systems and their associated neural circuits.^3^, ^4^, ^19^, ^53^, ^54^, ^77^ Empirical support for the involvement of various brain networks in creativity is primarily derived from neuroimaging studies.^20^, ^25^, ^33^, ^35^, ^78^, ^79^, ^80^

These networks conceptualize creativity as the ability to activate remotely represented ideas and concepts.^81^ In **Mednick**'s^82^ *flat associative hierarchy* model, this activation process concerns the structural organization of long‐term semantic memory networks, which is the type of semantic retrieval wielded when accessing one's knowledge stores, as defined in **Mendelsohn**'s^83^ *defocused attention* model and levels of trait *cognitive disinhibition*. Central to all these conceptualizations is the significance of *loosened associational thinking*, which is purportedly brought about by different information processing biases.^84^, ^85^ Given that the first two mechanisms—*defocused attention* and *flat associative hierarchies*—are focused on representation and access to conceptual knowledge, semantic cognition brain network regions would be implicated in information processing.

**FIGURE 3:**
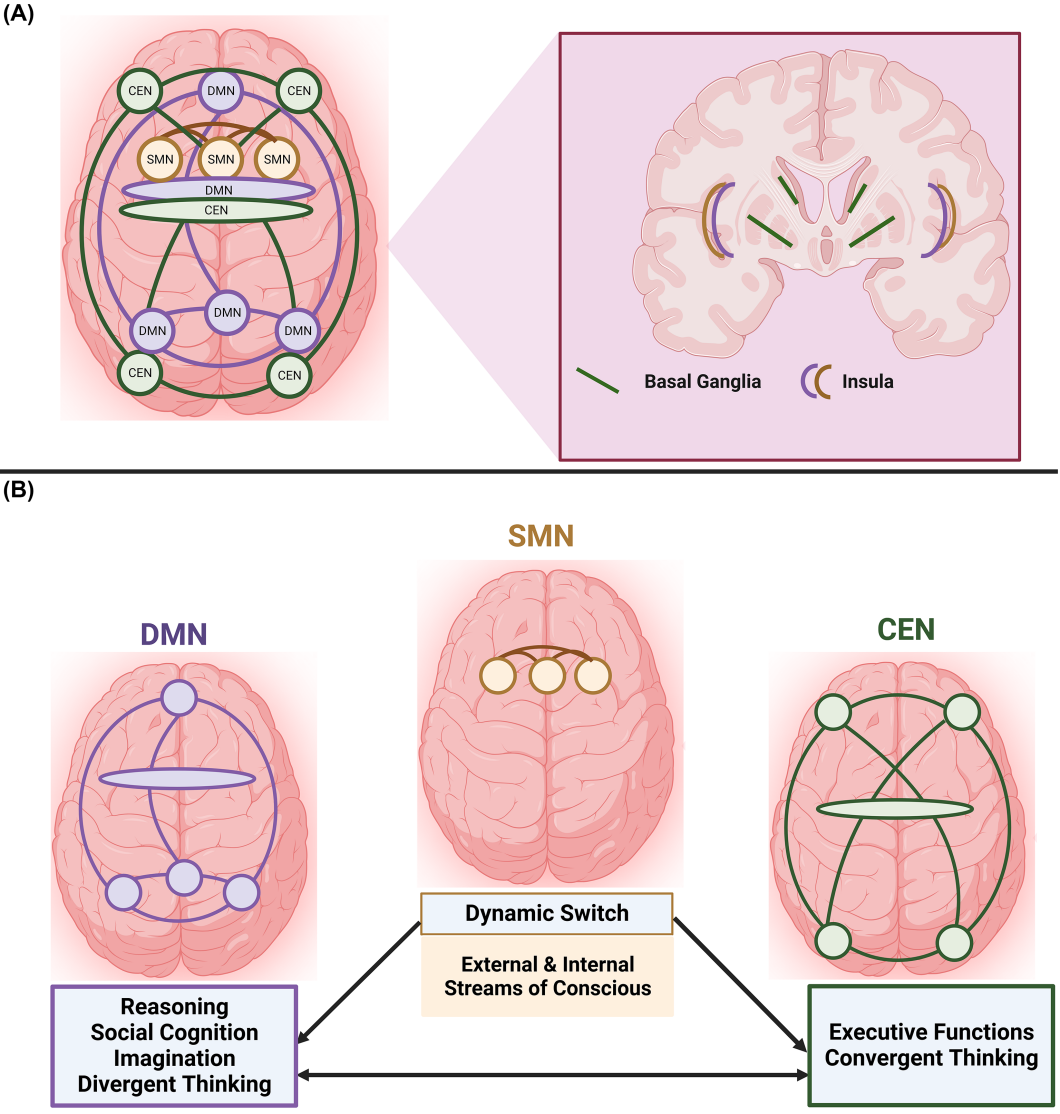
Schematic overview of the three creative network models. (A) Localization of the default mode network (DMN, purple), the salience mode network (SMN, brown), and the central executive network (CEN, green); and (B) The fundamental functions of each network model, which are described in the boxes below each model. The arrows refer to the interactions between these models based on their functions.

Brain areas that underlie the representation of multimodal conceptual knowledge (anterior temporal lobe or temporal pole), the controlled retrieval of conceptual knowledge (ventrolateral PFC or inferior frontal gyrus), or both (posterior lateral middle temporal gyrus), would be especially relevant for these network models.^86^, ^87^, ^88^, ^89^ It has been argued that there are three most frequently used networks,^25^, ^78^, ^79^, ^90^, ^91^ as shown in Figure 3.

The first network is CEN, which is activated when intensive attention and strong demands on working memory are required during creative thinking.^25^, ^78^, ^79^, ^92^, ^93^ The CEN is established in the lateral regions of the PFC and other dorsal brain areas; it is also implicated in the disinhibition mechanisms; therefore, regions critical in orchestrating inhibitory processes fall within the frontostriatal tracts and include the basal ganglia and dorsolateral and ventrolateral aspects of the PFC.^94^, ^95^, ^96^, ^97^, ^98^

The second network is the DMN (also called the imagination network and involved in social cognition, where the deep insula, PFC, and temporal lobe communicate with the parietal lobe). The DMN is primarily discussed in the context of reasoning about the perception, cognition, or behavior of one's self and others.^99^ Nevertheless, there is no doubt that it also plays a role in non‐social and non‐personal components of imaginative thinking, such as future semantic thinking^51^ and counterfactual reasoning.^100^ For these reasons, its significance in mental processes related to intentionality‐based imagination and innovative combinatorial imagination has been indicated in constructing dynamic mental simulations about imagining alternative perspectives and scenarios.^25^, ^78^, ^101^, ^102^, ^103^

The third network is the salience mode network (SMN), which constantly monitors both external and internal streams of consciousness; it is most salient for a particular task and is defined by two key nodes—the dorsal anterior cingulate cortex and the anterior insula.^33^, ^78^, ^79^, ^80^, ^92^ The right anterior temporal lobe, which comprises portions of the superior and middle temporal gyri, is considered a crucial brain region in the context of insight, and it is classified as one of the fundamental regions of the SMN and DMN.^104^, ^105^

These three networks share commonalities regarding the kind of information processing biases related to creative expression.^34^, ^35^, ^68^, ^80^, ^106^ However, CEN is involved in the closed, convergent, evaluative mode of creativity; in contrast, DMN is thought to be involved in the open, divergent, generative mode of creativity.^25^, ^33^

### 2.3 The mental workspace

Deciding whether a particular product, event, concept, action, etc., is “creative” means performing an estimate in a specific knowledge space, so a definitive decision might still be uncertain. The mental tools for estimating the value of a new idea or product are subjective and vary across contexts.^2^ Philosophers and scientists have long questioned where the human imagination comes from—what are the creative drivers behind the incredibly distinct behaviors of extraordinary scientists, artists, musicians, dancers, etc.?^2^, ^3^, ^4^ Imagination is one of the ultimate operators to enhance our creativity; thus, nurturing the activation of imagination, particularly in nature, is crucial. Because nature is ever‐changing, it provides countless opportunities and directions for exploring and reconfiguring the mental space,^3^, ^4^, ^107^ eventually boosting creativity.

Numerous studies have emphasized the correlations between exposure to natural environments and cognitive benefits, physical health, and well‐being. ^108^, ^109^, ^110^, ^111^ One potential mechanism for the benefit of spending time in nature is the perception of low‐level features of natural environments.^109^ More precisely, the visual domain's edge density has been demonstrated to convey semantic information and influence executive functions (EFs, i.e., cognitive inhibition, attention, memory, planning, cognitive flexibility, etc.; for a review, see^112^). According to White et al.,^110^ their study indicated that a minimum of 120 minutes per week spent in natural environments might serve as a crucial threshold for promoting health and well‐being among a diverse group of adults residing in England. However, additional investigations are demanded in this domain.

The ability to consciously change mental images is at the heart of many creative and uniquely human skills. Here, the question arises about how the human brain mediates such flexible mental operations. An interesting study by the Dartmouth Center for Health Care Delivery Science^107^ used functional magnetic resonance imaging (fMRI) to quantify how humans manipulate mental imagery. This study used a widespread neural network—the brain's “mental workspace,” which deliberately operates images, symbols, ideas, and theories and gives humans the mental focus to solve complex problems and express novel ideas—to identify 11 brain areas that showed differential activity levels. In a follow‐up study by Schlegel et al.,^63^ they conducted a subsequent investigation that introduced innovative multivariate techniques for examining the transmission of information within the cognitive domain while performing visual imagery manipulation. This study concluded that generating mental imagery requires disseminating information across various cortical regions and establishing shared representations.^113^

### 2.4 Emotion, cognition and flow: deliberate versus spontaneous modes

**FIGURE 4:**
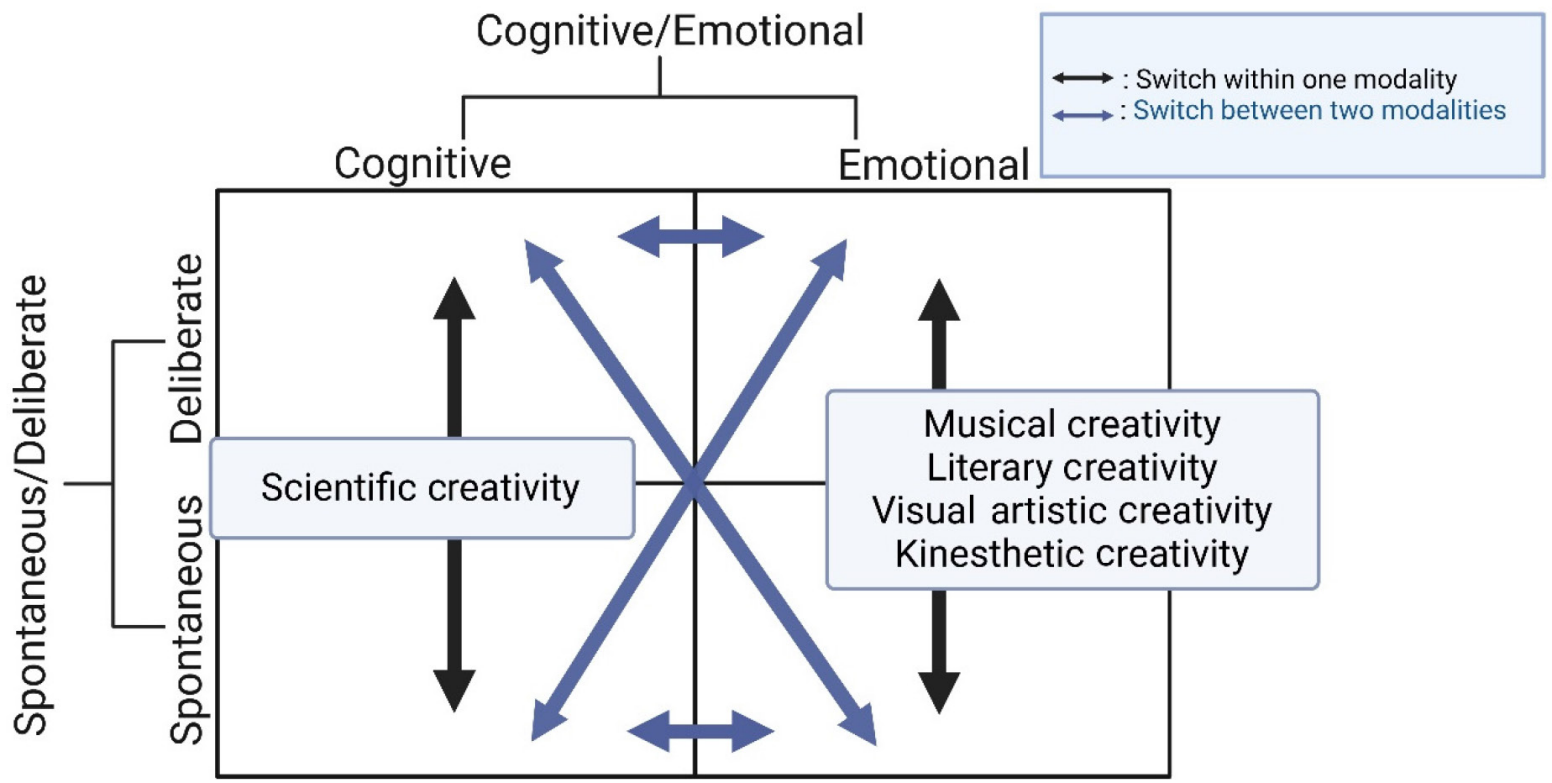
Four types of creativity with corresponding brain activities, according to Dietrich.^19^ We hypothesized a switch between two modalities (blue arrows) and within one modality (black arrows). Dietrich^19^ identified four types of creativity, either emotionally or cognitively based and also spontaneous (i.e., unexpected) or deliberate (i.e., a conscious effort to sustain), which results in four quadrants, each with different and unique aspects. We suggest different kinds of creativity for each quadrant: either cognitive (scientific creativity) or emotional (musical, literary, visual artistic, and kinesthetic creativity); however, there is a degree to which these types of creativity could change based on the switch between two modalities (blue arrows) and within one modality (black arrows).

Nineteen years ago, an influential account of different types of creativity argued for distinguishing between spontaneous and deliberate modes.^19^ According to this view, there are four types of creativity with corresponding brain activities, which can either be emotionally or cognitively based and spontaneous (i.e., unexpected) or deliberate (i.e., a conscious effort to sustain), resulting in four quadrants, each with different and unique aspects. It is necessary to distinguish between laboratory and realistic creativity in different domains. Under laboratory conditions, it is clear that we mainly assess deliberate modes of creativity; in contrast, spontaneous forms of creativity are too transient and unpredictable to be tested validly or reliably in controlled laboratory settings.^2^, ^20^, ^114^ Accordingly, we suggest that different kinds of creativity could be classified for each quadrant: either cognitive (scientific creativity) or emotional (musical, literary, visual artistic, and kinesthetic creativity), with a degree to which these types of creativity could change based on the switch between two modalities, within one modality, or both, as shown in Figure 4.

Establishing categorization and classification requires further investigation into employing various forms of creativity, focusing on the two modes and the cognitive and emotional elements.

## 3 BRAIN PLASTICITY AND CREATIVITY

Despite the distinctiveness of the previous models and views in explaining the information‐processing mechanisms underpinning creativity, the underlying brain plasticity (particularly metaplasticity and synaptic plasticity) is underestimated. What creative neural plasticity is and how intrinsic and extrinsic drivers are mentally processed to promote optimal creative outputs are unknown. Thus, approaching the mental operations that underpin creativity from a plasticity perspective will allow us to scrutinize these fascinating capabilities and investigate how neurons construct specific and distinctive pathways that allow this high degree of adaptability.

The term “brain plasticity” (also known as “neuroplasticity” or “cortical mapping”) refers to changes in the brain's neural pathways, which occur at the macroscopic, mesoscopic, or microscopic (synaptic) level and develop as a function of alterations in behavior (e.g., training‐induced), response to the environment (e.g., enriched, impoverished), and physiological processes (e.g., maturation, brain insults).^115^, ^116^, ^117^, ^118^, ^119^, ^120^, ^121^, ^122^, ^123^, ^124^, ^125^, ^126^ Brain plasticity opposes the idea that brain functions are fixed at a particular time^117^, ^127^; instead, it refers to the ability of the human brain to change as a result of one's experience that the human brain is “plastic” and “flexible”.^128^

Musical training, improvisation, and exercises are among the most effective inducers of brain plasticity and lead to improved EFs (in the short and long term, based on durations and intensities). In musical improvisation, fMRI studies have shown motor and auditory cortex reorganization in professional musicians; other studies revealed changes in neurotransmitter and hormone serum levels in correlation to music (for an overview, see^2^, ^129^). There was also a corresponding decrease in frontal beta EEG brain activity# after one hour of running, and this pattern remained stable for the remaining 5 hours.^130^ A reduction of beta waves following exercise is argued to constitute supportive evidence for the *transient hypofrontality hypothesis of flow*.^18^ “Flow” refers to the state of absolute contentment accompanying the experience when it occurs, that is, the absence of a clear sense of time when having a “peak experience.” Therefore, flow is experienced when an individual's complete focus is engrossed in an activity that aligns with their skill levels and the requirements of the task at hand.^131^, ^132^ In this state, individuals do not experience negative emotions or fatigue despite exerting effort to maintain optimal performance. Accordingly, most research on flow experience is conducted in physical activities that involve moving the body, like sports, exercise, and dance.^133^

The hypothesis of *transient hypofrontality of flow* is among the most prominent theoretical frameworks to explain flow experience, emphasizing the critical role of implicit and unconscious information processing and neural systems in facilitating the experience of flow, which is believed to occur due to a temporary weakening of cognitive control as orchestrated by the frontal lobe over other cognitive and neural systems.^18^, ^56^, ^134^, ^135^, ^136^

The following subsections will discuss whether experience flow varies among creativity domains, the underlying plasticity, and the relationship between creativity, risk‐taking, mental illness, and substance abuse.

### 3.1 Musical, visuospatial and Kinesthetic creativity

Philosophers, scholars, and scientists have long argued about the origin of the human imagination and the creative drivers behind the incredibly distinct behaviors of extraordinary scientists, artists, musicians, dancers, etc.^2^, ^3^, ^4^ We know that the human brain has information‐processing networks that facilitate the functioning of a mental workspace, thereby enabling the manifestation of high‐order creative and cognitive abilities. So in what manner does the information flow within these networks facilitate mental operations of creativity, and are they the same across creativity domains? To address these questions, we discuss brain plasticity across three creativity domains.

The power of music as an influential social bond in facilitating collective identity and a sense of community has been noted across diverse academic disciplines.^137^, ^138^ The apparent connection between music and enhanced performance or changes in neuropsychological activity has been demonstrated by studies involving Mozart's music, from which *The Mozart Effect* *theory* was derived. ^3^, ^4^, ^139^ In terms of music, the brain's plasticity in response to behavioral training in music^140^, ^141^, ^142^ and biofeedback training^143^, ^144^ has been highlighted.

Musicians from genres more typically associated with musical creativity, such as jazz, reveal a more comprehensive creative profile due to the power of improvisation in their practice.^145^ In a study that compared the musical activities of classical, folk, and jazz music students, those studying jazz accomplished more creative achievements and exhibited a greater frequency of engaging in extracurricular musical activities.^146^ Jazz musicians also exhibit greater openness to experience, a personality trait central to creativity, and better ideational originality in DT.^146^

Musical training and improvisation induce brain plasticity, but could neurological alterations (such as brain injury) result in the emergence of musical skills? Brain images of individuals when they listen to music show activity in different brain parts^142^; in particular, idiosyncrasies have been found in musicians' brain areas, including the motor cortex, the cerebellum, and the corpus callosum. Various other studies have indicated that listening to music can improve cognition, motor skills, and recovery after brain injury (for a review, see^3^, ^129^). Generative processes are held to be inherent to music performance, music composition, and musical improvisation.^147^, ^148^, ^149^ However, the limited evidence that has examined whether playing music suffices to foster greater creativity has indicated that this is not necessarily the case. For example, only expert musicians who create music through composition or improvisation generate more creative uses for musical items; this differs from non‐musicians or expert musicians who do not create music.^150^

On the other side of the coin are disorders of music perception.^151^, ^152^, ^153^ A strong tendency toward musicality has been distinguished in atypical populations, such as those with autism spectrum disorders (ASDs)^154^, which is to some extent counterintuitive, given that the primary deficit of ASDs is severe impairments in communication and social interaction (for a review, see^155^). Therefore, there is growing evidence of the utility of music therapy in ASDs rehabilitation for improving verbal and non‐verbal communication skills and socio‐emotional reciprocity.^156^ Nevertheless, the precise mechanisms underlying such interactions are unclear due to the intricate relations between richly intertwined and interconnected brain networks. Thereby, evaluating the nature of the relationship between music and brain plasticity in healthy and atypical individuals and its broader impact calls for considerable investigation since there are various pertinent issues to address.

Studying blind artists has demonstrated the sharp power of the brain's plasticity in the context of the creation of visual art. At this juncture, extreme neural plasticity occurs, which refers to the capacity of the brain to continually reorganize itself as a function of experience by building new neural connections.^157^ One fascinating case study showed the brain's ability to generate recognizable images of an object after exploring it through touch^158^ and drawing, whereas the brain engages in visual processing regions, including the primary visual cortex (V1).^159^ Even training a congenitally blind person to draw can result in a rapid cortical reorganization, as indicated by the novel recruitment of V1 (which, before training, displayed an undifferentiated response for a non‐visual task).^160^

These remarkable examples shed light on the possibility of exploring in greater depth what we know about the psychological and neural underpinnings of visual artistic creativity and how these are further distinguished as a function of artistic expertise. Moreover, researchers in the field of rehabilitation who subscribe to the empowerment and inclusion paradigm may contemplate using the benefits of visual arts‐based research to achieve their goals.^113^, ^161^ Consequently, visual arts‐based research can have the additional effects of empowerment interventions, and they may be a foundation for developing and refining formal best practices for this emerging research approach.^162^

Unlike verbal or visuospatial creativity, kinesthetic forms of dance and sports involve sensations of whole‐body movement, or “kinesthesia,” which refers to the integration of information from the vestibular system (the sensory experience of balance and spatial orientation) and the proprioceptive system (the sensory experience of forces within the body's muscles, tendons, and joints).^2^ Some accounts of kinesthesia include proprioception and exteroception (the sensory experience—visual, auditory, and tactile—from stimuli outside the body), making kinesthesia integral to multisensory and active perception.^163^

The *transient hypofrontality hypothesis of flow* is further elaborated upon concerning performance‐based creativity. Therefore, a multiple‐factor model that adopts evolutionary perspectives and focuses explicitly on forms of creativity akin to kinesthetic creativity can assist in specifying the detailed mechanisms that lead to creative responses in such contexts. Trust and confidence in exerting exquisite control over one's bodily movement are crucial elements that cannot be ignored in this context.^2^, ^163^

Achieving optimal performance in physical activities requires precise timing, rhythm, and synchronicity in movements throughout the entire body. The proximal conditions in these situations can be easily determined, and the phenomenon of flow experience is positively correlated with actual superior performance.^18^, ^131^, ^132^, ^164^ Some studies have proposed that activities involving the entire body's movement are more favorable for inducing flow experiences than other activities.^133^, ^165^, ^166^ Therefore, dancers or athletes tend to indicate greater levels of dispositional flow than opera singers.^167^

**FIGURE 5:**
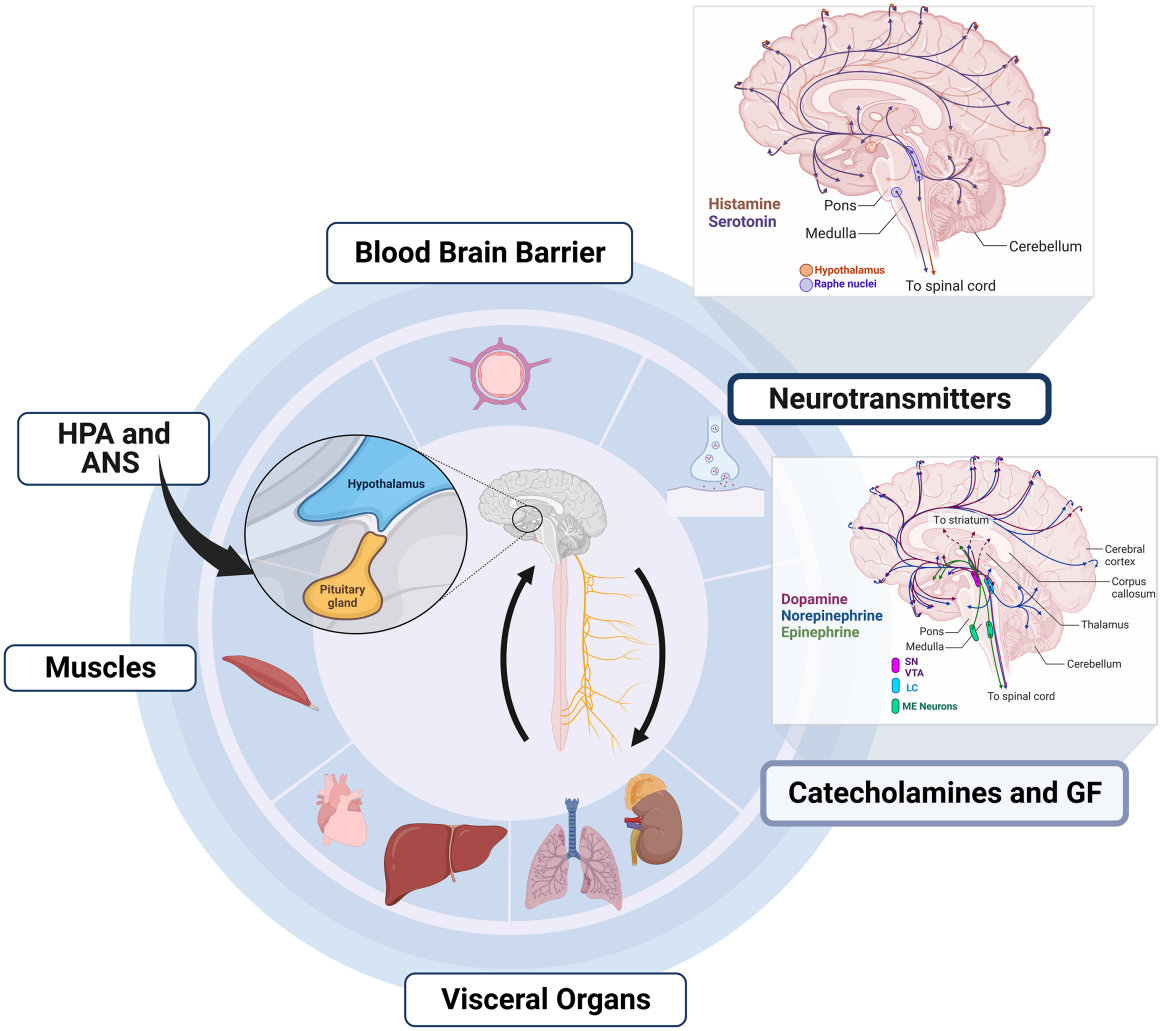
Diagram of the components and processes that induce brain plasticity through physical activity. HPA and ANS refer to the hypothalamic–pituitary–adrenal axis and autonomic nervous system, respectively. HPA and ANS represent the machinery for converting the energy from visceral organs to the brain regarding catecholamines (i.e., dopamine, norepinephrine, and epinephrine), GF (growth factors), and neurotransmitters (i.e., histamine and serotonin). Brain illustrations depict the distribution of neurotransmitters and catecholamines.

Regarding brain plasticity in the context of kinesthetic creativity, Murillo‐Garcia et al.^168^ conducted a systematic review demonstrating the significant impact of dance‐based interventions in reducing the effects of fibromyalgia and pain. The subgroup analyses have indicated that creative dance‐based interventions may have greater efficacy than repetitive interventions in reducing the effects of fibromyalgia and pain. Given the significant heterogeneity and limited number of articles, caution must be taken when interpreting the results. Moreover, physical exercise has positively influenced EFs and neuroplasticity,^169^, ^170^, ^171^, ^174^ particularly in aging populations.^172^, ^173^ The reasons for improving EFs induced by regular exercise seem to be facilitated by the interaction between the brain, sympathetic, and parasympathetic systems that influence regional cerebral blood flow and the production and release of neurotransmitters and psychological factors (Figure 5).

**FIGURE 6:**
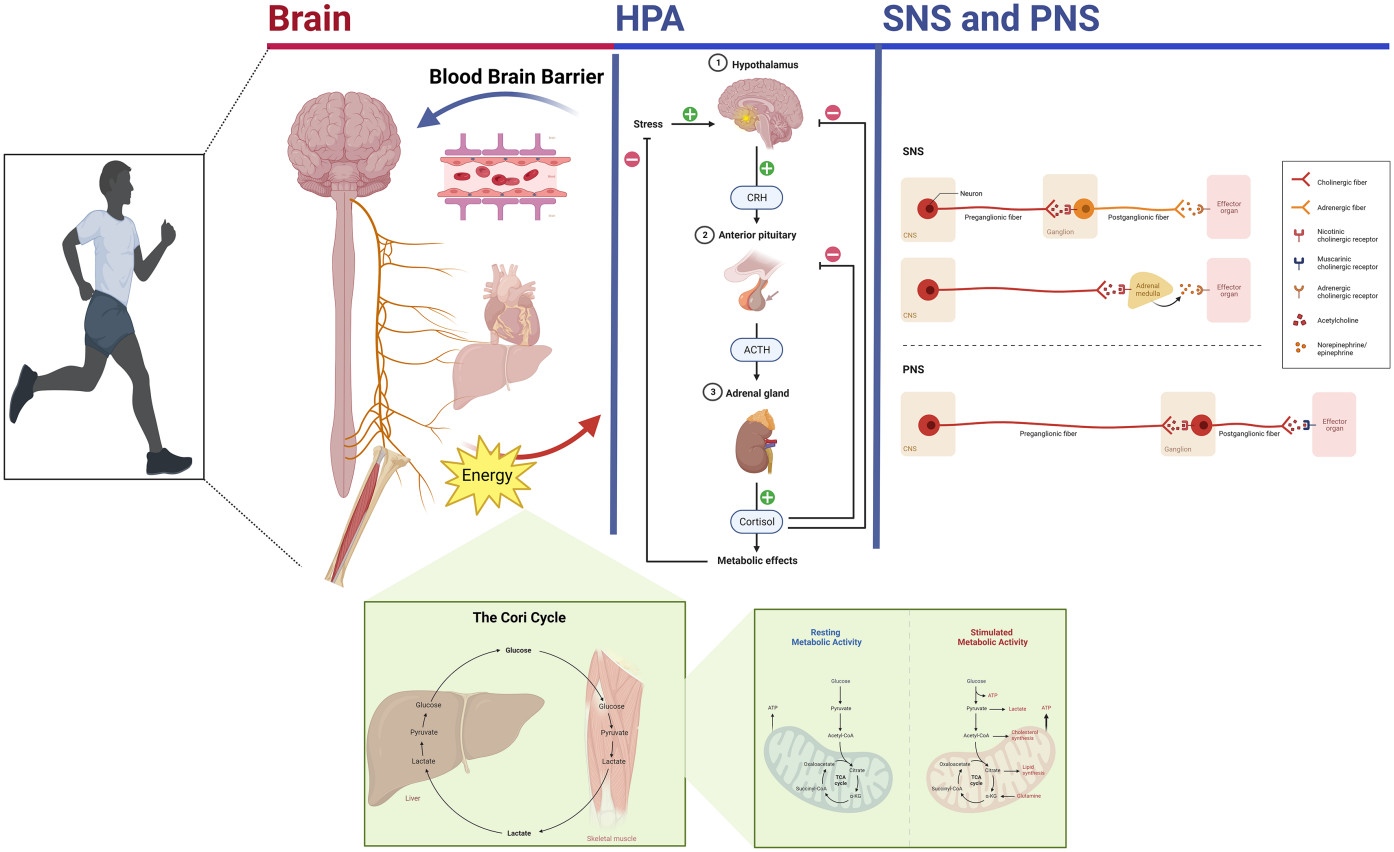
Graphic illustration depicting how physical activity affects the brain, the hypothalamic–pituitary–adrenal axis (HPA), and the sympathetic and parasympathetic nervous systems (SNS and PNS). The brain and visceral organs, such as the liver, heart, and lung, convert energy into neurotransmitters, which the blood–brain barrier uptakes. HPA reduces stress. SNA and PNS have different organizational machinery for signal transduction. The Cori cycle (interaction between the liver and muscle for cycling glucose and lactate) is shown in the lower graph (metabolic activities in the mitochondria during rest (blue) and exercise (i.e., active, which involve the release of ATP, red)).

Zooming in on the neurobiological mechanisms of the positive influence of exercise concerning human physiology is essential to better understand the phenomenon of the flow of experience related to kinesthetic creativity. The five most extensively studied key neurobiological pathways supporting physical exercise have been assembled in a review by Chen and Nakagawa.^173^ According to this review, five mechanisms explain how enhancing exercise performance and the associated body's adaptations benefit the brain by enhancing EFs. These five mechanisms require interaction between the brain, sympathetic, and parasympathetic nervous systems (Figure 6).

One of the mechanisms involves releasing growth factors necessary for neurogenesis and angiogenesis (i.e., the development of neurons and blood vessels). The second mechanism entails lactate synthesis, which supplies the brain with energy, generates glutamate, and preserves long‐term potentiation. The third mechanism depends on the release of anti‐inflammatory cytokines that reduce neuroinflammation. The fourth mechanism includes increased mitochondrial biogenesis and antioxidant enzyme activity that lower oxidative stress. The final mechanism implicates the release of neurotransmitters such as dopamine and serotonin that control neurogenesis and affect cognition. Thus, the type and intensity of physical activity (i.e., duration, etc.) determine the magnitude of the positive effects on EFs, which should also be considered when physically engaged in exercise.^173^, ^174^

Despite these fascinating positive influences of physical exercises on inducing brain plasticity, their impacts on creative thinking are still mixed, and further research is required in this framework. For instance, improved positive mood and ideational flexibility have been reported following a brief aerobic workout.^175^ However, factors like athletic expertise, the intensity of exercise, and the type of creativity measurement also need to be evaluated. One study by Colzato et al.^176^ showed that non‐athletes exhibit poorer convergence of creativity under intense exercise than moderate exercise or rest. In contrast, athletes' convergent thinking (CT) was not significantly different across the three conditions. Moreover, athleticism did not affect DT, but ideational flexibility was superior under rest conditions than intense exercise across all participants. The benefits of simple physical activity such as walking vary relative to the creativity measure; such activity improved ideational originality performance in 81% of individuals with DT but only 23% for CT.^177^

**FIGURE 7:**
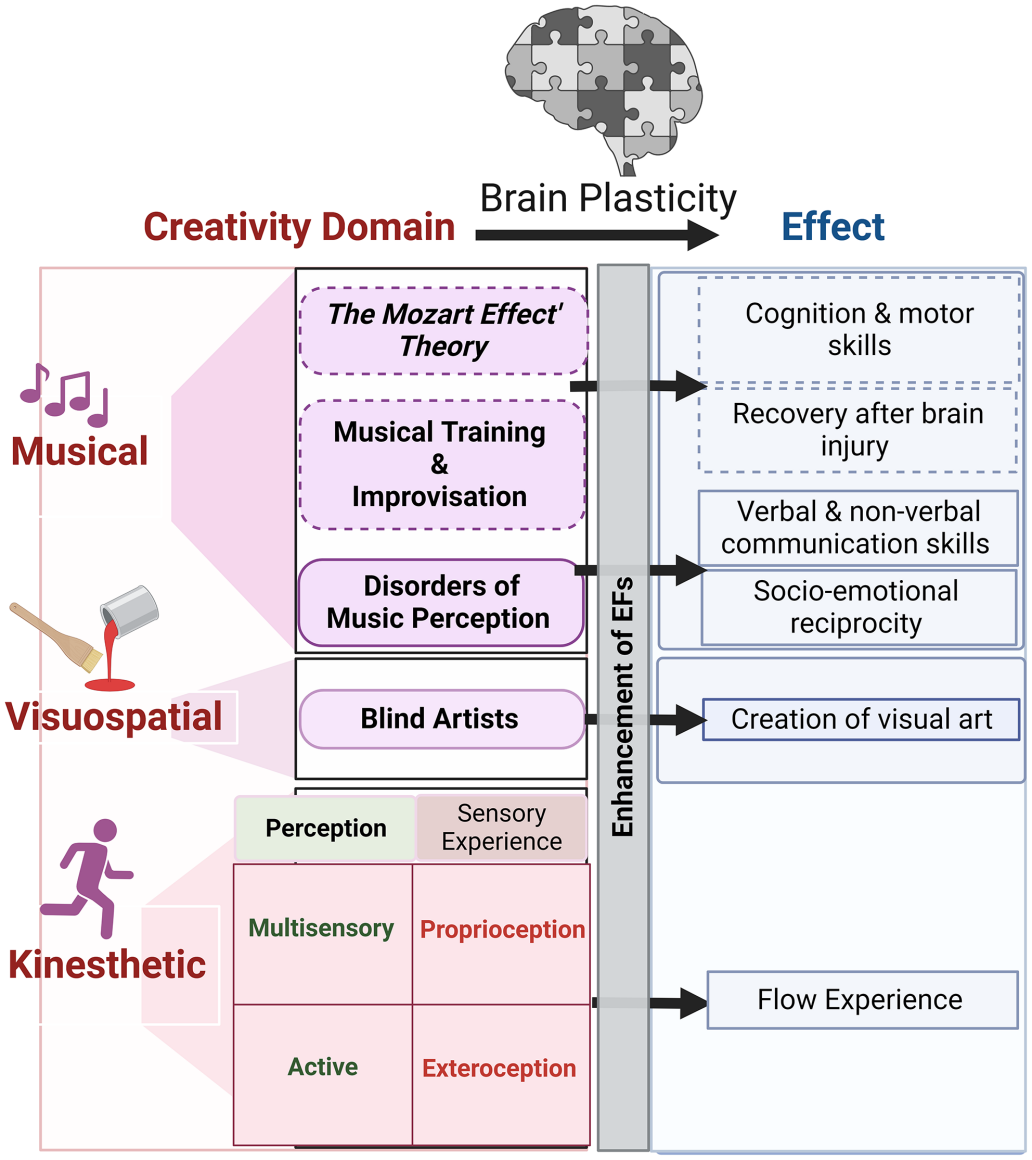
Graphic depiction showing how three creative domains—musical, visuospatial, and kinesthetic—affect brain plasticity in individuals who have suffered brain injuries. The distinct plasticity effect appears in the three creativity domains through enhancing executive functions (EFs), each indicated by arrows.

Consequently, future research agendas on effective creative therapy should consider all these elements across the different creativity domains when implementing experimental designs. The power of brain plasticity in improving EFs across three types of creativity is summarized in Figure 7.

### 3.2 The tie between creativity and risk‐taking, mental illness and substance abuse: fact or fiction?

In an excellent review by **Heilman** ^178^ on the possible mechanisms of creativity, it is pointed out that creative individuals are often risk‐takers and novelty seekers—the behaviors that are associated with activating the ventral striatal reward neural system; however, the correlation between risk‐taking and creativity has been insufficiently explored. Tyagi et al.^179^ conducted an extensive systematic review of this connection, examining the relationships between creativity and five distinct types of risk‐taking (financial, health and safety, recreational, ethical, and social). The findings of the offline study revealed that creativity is linked to a strong propensity for risk‐taking in the social domain rather than in other areas. Indeed, the online study, with a larger sample size and a more diverse population, revealed that the likelihood of social risk‐taking was the most effective predictor of creative personality and ideation scores.

When it comes to musicians and risk‐taking, other considerations should be taken into account. Here are the two sides of the coin. The first side is that while it is well known that musicians strive for flawless performances and perfection, avoiding errors at all costs, the other side is that musicians must be willing to take risks and potentially make errors to achieve innovation or exceptional performance.^180^ Coping with errors during practice or performance can be a source of frustration, and the experience of anger and despair may account for the generally unfavorable disposition of musicians toward errors and their inclination to strive for flawless learning in the context of instrumental music education.^180^

These results pointed to the need for a more multifaceted conceptualization of creativity and other related cognitive processes and for treating creativity and risk‐taking as multidimensional attributes. This calls for more studies using EEG and fMRI to determine the brain structures linked to risk‐taking tendencies during creative performance across different domains.

The concept that creativity and mental illness are strongly related and have a long history has been advocated from multiple perspectives (e.g., psychoanalytic, cognitive, clinical, genetic, and evolutionary^181^, ^182^, ^183^, ^184^). The guiding rationale is “paradoxical functional facilitation,” where direct or indirect neural deficiencies can facilitate psychological functions.^185^ Although the field of neuroscience of creativity remains polarized on the nature of the link between creativity and mental illness,^2^, ^101^ with some scholars even questioning its validity,^186^, ^187^, ^188^ the empirical basis of this association is mainly derived from the raised incidence of severe mental illness, particularly bipolar disorder, and schizophrenia, among eminent and high achievers in creative professions.^189^, ^190^, ^191^, ^192^, ^194^

Along with circumstantial accounts, much empirical evidence (mainly from psychology) suggests an association—even if modest and complex—between mental illness and creativity.^101^, ^195^ In contrast, there is growing evidence for the opposite association, particularly from the public or community mental health discipline, which indicates that engaging in creative pursuits improves mental well‐being.^196^, ^197^ This notion is the logic behind using art therapies to manage various health‐relevant conditions.^198^ Although it appears counterintuitive that both of these ideas can co‐exist, this may be possible as they reflect various aspects of the same associative link and refer to several levels of functionality.^199^

The associations between specific forms of mental illness and a high inclination for creativity are pervasive. The higher prevalence of mental illness in people who work in creative professions, such as writers and artists, primarily informs this premise.^190^ Many empirical studies have also examined how creative thinking is associated with specific forms of mental illness, such as schizophrenia and bipolar disorder, and their subclinical variants.^101^, ^195^, ^200^ The rationale for explaining the apparent advantages of specific aspects of creative cognition for such groups comes from claims that biased information processing (e.g., cognitive disinhibition or overinclusive thinking) usually leads to insufficiencies in goal‐directed thought and action in some contexts (i.e., negative biases). These negative biases can lead to particular advantages in open‐ended contexts that call for creativity in thought and action (i.e., positive biases). There is ample literature on the tie between mental illness, substance abuse, and creativity (especially literary creativity) that is relevant to the questions posed when studying the neuroscience of these issues. Creative writers have been singled out as having a propensity toward mental illness and substance abuse.^189^, ^193^, ^201^, ^202^, ^203^, ^204^

There are several open questions about the nature of the association between creativity and mental illness, three of which are highlighted here. The first is the shape of the association, with some researchers arguing for an inverted‐U function of sorts (e.g.,^32^, ^205^, ^206^). The second concerns integrating what we know about the link between creativity and mental illness; some literature, for example, suggests that creativity is positively related to mental well‐being. The third question is about what comes first, creativity or mental illness. It is particularly relevant in the context of vulnerability to substance abuse, the use of psychostimulants,* and the cultural context of use and creative achievement.^207^

Recent reports did not reveal a significant effect of different types of psychostimulants on DT and CT in healthy^208^ and unhealthy (i.e., attention‐deficit/hyperactivity disorder; ADHD) populations.^209^ The study by Baas et al.^208^ reported on methylphenidate as a cognitive enhancer, and the results did not indicate any effects on divergent or convergent creative processes, regardless of individual differences in working memory capacity or impulsivity. Hoogman et al.^209^ reviewed 31 behavioral studies examining the relationship between creativity and ADHD. This review distinguished between various research designs, age groups, creativity measurements, and psychostimulants' effects, reflecting the potential underlying neural mechanisms of creativity and ADHD. Most studies found evidences for increased DT in those with high ADHD scores (subclinical) but not in those with the disorder (clinical). The creative abilities and achievement rates were high among clinical and subclinical groups. There was no evidence for increased CT abilities in ADHD, nor did the analyses indicate an overall negative effect of psychostimulants on creativity.

Given the scarcity of research and investigations regarding the correlation between psychostimulants, mental disorders, and creativity, more research is required to formulate more comprehensive and concrete conclusions. Conducting more research on creative professionals across various domains is imperative to establish precise links. The current literature does not provide a definitive conclusion, and excluding creative professionals from the study may hinder accuracy.

### 3.3 Awakening creative production and enriching the creative state of mind in Dementia patients

The empirical root for the view that reduced top–down or concept‐driven processing leads to enhanced creativity emerges from various sources. The most compelling of these sources is the study of individuals with savant skills. These skills are defined as having exceptional or prodigious capabilities in at least one domain (e.g., art, music, speed calculation, memory) far exceeding any typical level. Around 10% of those with savant skills are classified as “acquired savants,” their skills suddenly manifest following a stroke,† dementia,‡ or head injury.§ Most are classified as “congenital savants,” their skills are visible very early during development; therefore, ASDs is overwhelmingly associated with this condition (estimated between 50 and 75%). Conversely, one in 10 people with ASDs also exhibits savant skills.^210^ Similarly, some studies included people with savant syndrome, whose savant skills persist despite considerable neurodevelopmental disorders or brain injuries.^211^, ^212^

Many fascinating case studies have examined the presence of savant skills alongside psychological deficits^213^; see Table 1.

**TABLE 1:** Some examples of savant skills, de novo artistic abilities in developmental savants, and psychological deficits in visuospatial and literal creativity.

Skill	Case	Creativity domain	Developmental savants/Psychological deficits
Savant skills	**Nadia Chomyn**	Visuospatial	Autistic savant
	**Stephen Wiltshire**		
	**Willem de Kooning**		Alzheimer's disease
	**Louis Corinth**		Visuospatial neglect after a right hemisphere stroke
	**Vincent van Gogh**		Psychosis, Substance Abuse, and Epilepsy
De novo *artistic* proficiency	Three patients became accomplished painters		The onset of FTD (temporal‐lobe variant)
	Three patients developed primary progressive aphasia or semantic dementia	Literal	Relative sparing of certain parts of the lateral temporal lobe inside the superior and middle temporal gyri. Severe atrophy of the temporal poles and medial temporal regions such as the amygdala, parahippocampal gyri, and limbic areas as the insula

A spectacular case is that of **Nadia Chomyn**, an autistic savant with profound language and social deficits who began drawing at the age of 3. While **Nadia** inexplicably lost her ability to draw before puberty, most experts do not exhibit this pattern. The aesthetic value of her sketches as a 7‐year‐old was deemed comparable to some of those by **Leonardo da Vinci**.^214^ A case in point is **Stephen Wiltshire**, whose extraordinary drafting skills show no signs of decline. There are numerous case studies of artists who have suffered altered brain function or experienced forms of brain damage.^215^, ^216^ Prominent examples include the artists **Willem de Kooning** (Alzheimer's disease), **Louis Corinth** (visuospatial neglect following a right hemisphere stroke), and **Vincent van Gogh** (psychosis, substance abuse, and epilepsy).^2^ While artists with brain deficits show a range of psychological deficits, such as memory retrieval and language production, they continue to produce art unobstructed unless their motor functions are directly affected.^3^, ^4^, ^129^ These remarkable states have been taken as evidence of the damage‐resistant capacity of the human brain and its power to communicate and express itself through art.^217^, ^218^

Music‐making and listening are used in neurorehabilitation to improve deficits in speech, motor function, and other psychological functions arising from brain disorders (i.e., following a stroke or with the onset of neurodegenerative disease). In this context, widely used techniques for neurorehabilitation include melodic intonation therapy, auditory‐motor mapping training, music‐support therapy, and auditory rhythmic movement. Creative arts‐based therapies are also used in poststroke rehabilitation to enhance the quality of life, and there is accumulating evidence of improvements in EFs, mood, and well‐being when using neurorehabilitation to treat the effects of normal aging.^129^, ^142^, ^219^, ^220^, ^221^, ^222^, ^223^ Different art modalities are perceived as helping patients achieve several therapeutic goals, and interventions that offer opportunities to experience other art modalities to enhance cognitive flexibility. As a result, more research is required to determine the differential benefits or particular advantages of using single or multiple art modalities in creative arts‐based therapies.

De novo artistic proficiency is another fascinating occurrence where artistic tendencies suddenly develop in non‐artists following brain injury, see Table 1. This de novo generative capacity often manifests in the visual and musical arts and is associated with various conditions, including frontotemporal dementia (FTD),¶ migraine, epilepsy, and traumatic brain injury.^228^ The single most common case studies of individuals with specific neurological damage have been examinations of neurological patients with specific forms of FTD who develop de novo *creative* abilities or individuals who have savant‐like capabilities.^211^, ^217^, ^224^, ^225^, ^226^, ^227^

In the first research study of its kind, Miller et al.^226^ reported on three patients who became accomplished painters following the onset of FTD. All three had the temporal‐lobe variant of FTD, whereby their frontal lobe was relatively intact but their temporal poles were dysfunctional. While the de novo emergence of artistic proficiency is a rare occurrence in these conditions and features in only a minority of cases (e.g., 17% of the 69 FTD patients reported in^225^), there are consistent behaviors across cases (e.g., compulsiveness of the need to paint) that deserve further elaborate analysis. It is also highly probable that the appearance of such features is underreported.^228^ Because most individuals beset with such conditions do not display enhanced musical skills, and because of the heterogeneity regarding the brain correlates of such disorders and the extensive networks involved,^229^ it is not yet possible to specify which regions of the brain are especially significant in the sudden emergence or unexpected presence of musical capacity.

Although brain injury causes flaws in certain facets of functioning (such as loss of semantic understanding, social awareness, or speech production), it can also result in enhanced de novo abilities relevant to visual art or music, as shown in the previously mentioned case studies of individuals with FTD.^217^, ^225^, ^226^ However, these cases represent a rare manifestation and are only seen in a subset of patients, such as those with FTD, where the degeneration of brain tissue affects the temporal lobe but not the frontal lobe. It is also crucial to remark that, in contrast to the case of savants, the skills shown in this context of neurodegenerative disorders are rarely extraordinary. Nevertheless, they are wholly unexpected, given the level of skills exhibited by the patient before the neurological insult. In such neurological cases, turning to art is innovative; the created art is not necessarily creative.^218^ The motivation or drive to communicate despite language and communication deficits in neurological disorders may explain such behaviors; thus, art provides an alternative form of expression and communication.^218^

Unlike visual art and music domain studies, literary domain studies rarely examine de novo creative emergence after a neurological insult. Among these rare studies is the case report by Wu et al.^230^ on literary creativity in three neurological patients following primary progressive aphasia or semantic dementia. Their brain scans revealed relative sparing of some parts of the lateral temporal lobe within the superior and middle temporal gyri alongside severe atrophy of the temporal poles and medial temporal regions, such as the amygdala and parahippocampal gyri, and limbic regions like the insula. ^230^

Some neuropsychologists question the vast utility of neuroimaging approaches to providing theoretically relevant insights about behavioral and brain functioning,^231^, ^232^ while others have identified specific contexts in which neuroscientific and neuropsychological approaches can be used together.^233^ We argue that combining both neuroscientific and neuropsychological approaches will enable us to progress our understanding of the psychological function of creativity in different domains concerning physiological factors of its mental operations in health and disease.

The discipline of neuropsychology aims to better understand the function of human psychology concerning physiology by examining dysfunctions of the brain.^178^, ^234^, ^235^, ^236^, ^237^ Such investigations can be undertaken through single‐case studies of individuals with specific neurological damage or group studies on populations that reflect specific neurological disorders or psychiatric conditions. In both situations, the guiding factor is that the psychological dysfunctions accompanying brain injuries or deficits are exceedingly informative about the healthy human brain. While the advantage of the neuropsychological approach is its ability to be theoretically informative, the disadvantage is its inability to fully control for the impact of co‐morbidities (emotional, motivational, motoric, etc.) that often accompany brain dysfunctions.

**FIGURE 8:**
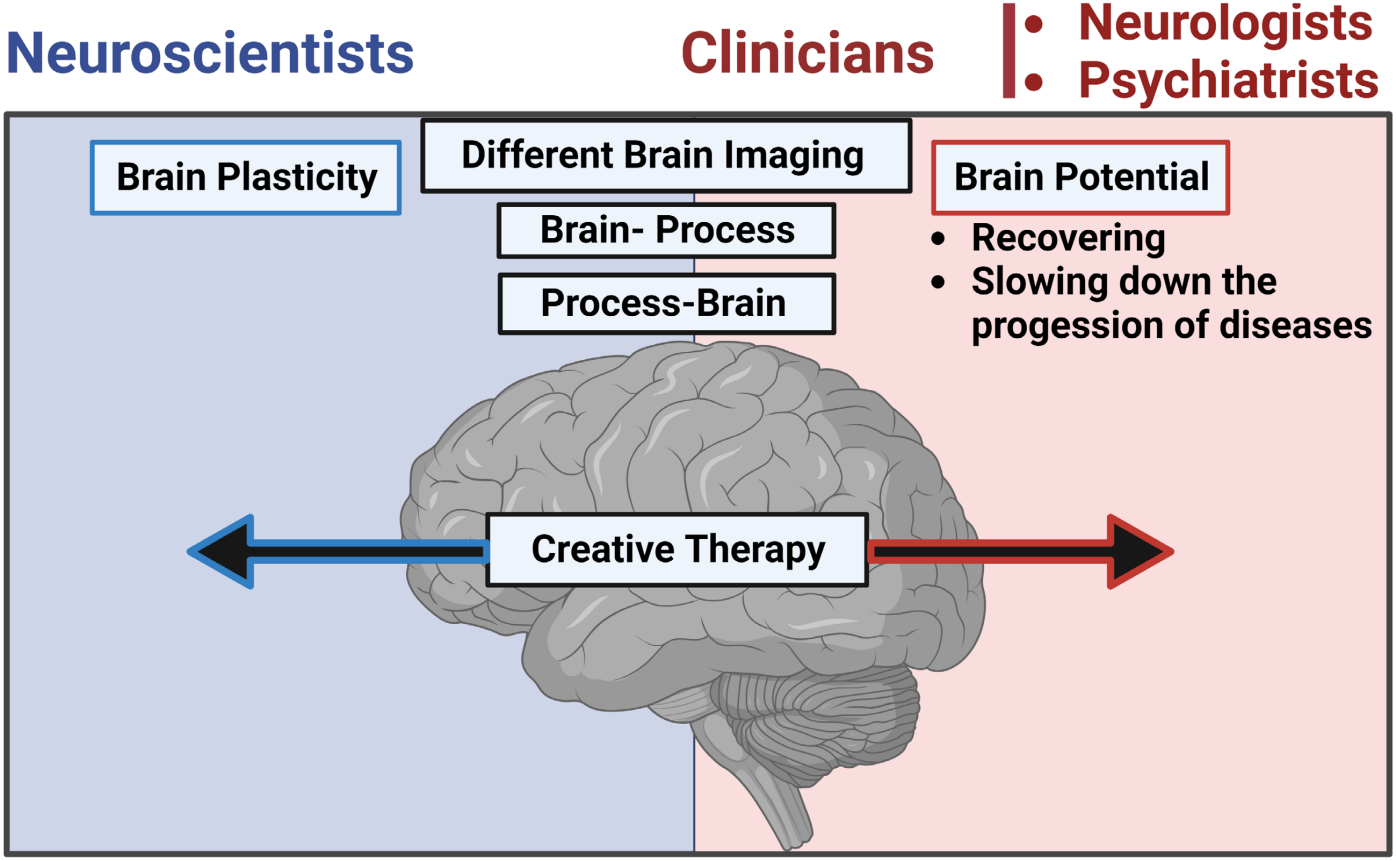
Illustration of neuroscientists' and clinicians' perspectives on creativity. Neuroscientists attempt to explain neuroplasticity using brain imaging techniques and brain‐process and process‐brain approaches. In contrast, clinicians (neurologists and psychiatrists) aim to find ways to promote the “brain potential” in recovering from or slowing the progression of diseases. Uniting the two communities will deliver more promising benefits from using creativity as an effective therapy for health and disease.

Neuroscientists use brain‐process and process‐brain approaches to explain neuroplasticity, whereas clinicians (mainly neurologists and psychiatrists) utilize these approaches to promote the “brain potential” in disease recovery or slowdown. Integrating the three specialties can provide precise explanations for using creativity as an effective intervention/therapy (Figure 8).

Therefore, it is imperative to examine the neural mechanisms of artists and other creative professionals who have experienced brain injuries. This will allow us to gain details from previous case studies employing integrative approaches. It is crucial to note that the mere acquisition of artistic skills, such as playing music or painting, does not necessarily qualify as a “creative” act. The distinction lies in the fact that the act of “creating something” is distinct from the act of “creating something that is creative.”^2^, ^52^, ^78^, ^79^, ^92^, ^114^, ^238^, ^239^, ^240^, ^241^

## 4 SUMMARY AND CONCLUDING REMARKS

This review provides a synopsis of the creative mind and the power of creative therapy, as viewed through the lens of neurobiology. This synopsis underlines several pivotal questions that still demand further exploration to implement creative therapy approaches effectively. The various challenges in studying creativity compared to other complex psychological functions lend themselves to objective scientific investigation; thus, the neuroscience of creativity needs up‐to‐date, empirically testable ideas that maintain ecological and biological validity and reveal underlying mechanisms. As most creativity tasks are not designed to tease apart such closely coupled cognitive capacities, one can draw only limited conclusions about the dynamics of the underlying mental operations. Therefore, the associated brain correlates have yet to be established and merit further investigation from a theoretical standpoint.

These reasons prompted us to reflect on the overlooked potentials of creativity in health and disease such as creative therapy and how it could positively and significantly improve our well‐being, providing a glimmer of hope, particularly for those suffering from neurodegenerative diseases. Here, we argue for implementing effective creative therapeutic approaches in different creativity domains (arts, music, sport, dance, etc.), which necessitates a comprehensive and integrative awareness of how our current knowledge from the psychological and neuroscientific literature informs us about the distinctive mechanisms of de novo abilities.

In light of the state of the art of research and literature on creativity from a neuroscience viewpoint, we are constrained, as the studies of brain plasticity underlying creativity require elaborate investigations. Hence, it is crucial to acknowledge these limitations to avoid overgeneralization and maintain a firm footing on the path of authentic and accurate exploration. Using examples from various creative disciplines, we discuss what future studies should focus on. Lastly, we highlight awakening creative production and enriching the state of mind in dementia patients, which opens the gate to properly contemplating creative therapy.

## CONFLICT OF INTEREST STATEMENT

The authors declare that this work has no conflict of interest.

## Data Availability

Data sharing not applicable to this article as no datasets were generated or analysed during the current study.

## Endnotes

# Beta brain waves indicate focused attention and alertness.

* Psychostimulants are a class of drugs that significantly impact cognitive and emotional functioning and behavior. Psychostimulants are conventionally limited to cocaine and amphetamines. Nicotine, caffeine, and selected antidepressants may be classified within this category. Methylphenidate, dextroamphetamine, and pemoline (the latter of which was withdrawn from the market due to hepatotoxicity) have been extensively utilized to control attention‐deficit/hyperactivity disorder (ADHD).

† A stroke occurs when a brain blood artery bursts or a blood vessel stops blood flow.

‡ Dementia is the inability to remember, think, or make decisions that interfere with daily life. Most people with dementia have Alzheimer's. Dementia typically affects elderly people.

§ Brain injury can manifest as either a closed or open (penetrating) injury to the head. A closed head injury refers to an instance where an individual has experienced severe damage to the head, without any accompanying fracture to the skull, resulting from contact with an object.

¶ A core feature of brain disorders such as savant syndrome.^210^, ^212^, ^225^, ^242^
